# Score Prediction of Sports Events Based on Parallel Self-Organizing Nonlinear Neural Network

**DOI:** 10.1155/2022/4882309

**Published:** 2022-01-15

**Authors:** Junyao Ling

**Affiliations:** Xi'an University of Finance and Economics, Xi'an 710100, Shaanxi, China

## Abstract

This paper introduces the basic concepts and main characteristics of parallel self-organizing networks and analyzes and predicts parallel self-organizing networks through neural networks and their hybrid models. First, we train and describe the law and development trend of the parallel self-organizing network through historical data of the parallel self-organizing network and then use the discovered law to predict the performance of the new data and compare it with its true value. Second, this paper takes the prediction and application of chaotic parallel self-organizing networks as the main research line and neural networks as the main research method. Based on the summary and analysis of traditional neural networks, it jumps out of inertial thinking and first proposes phase space. Reconstruction parameters and neural network structure parameters are unified and optimized, and then, the idea of dividing the phase space into multiple subspaces is proposed. The multi-neural network method is adopted to track and predict the local trajectory of the chaotic attractor in the subspace with high precision to improve overall forecasting performance. During the experiment, short-term and longer-term prediction experiments were performed on the chaotic parallel self-organizing network. The results show that not only the accuracy of the simulation results is greatly improved but also the prediction performance of the real data observed in reality is also greatly improved. When predicting the parallel self-organizing network, the minimum error of the self-organizing difference model is 0.3691, and the minimum error of the self-organizing autoregressive neural network is 0.008, and neural network minimum error is 0.0081. In the parallel self-organizing network prediction of sports event scores, the errors of the above models are 0.0174, 0.0081, 0.0135, and 0.0381, respectively.

## 1. Introduction

With the mature application of neural network technology and the rapid development of Internet, the research of data prediction has emerged. Data prediction often faces some noisy, messy, and nonlinear data. Neural networks have good robustness, [1]. This article briefly introduces the main clustering algorithms in data prediction, conducts an in-depth study, and optimizes the SOM learning algorithm, which improves the network iteration to a certain extent. The speed of training layer expansion self-organizing mapping network (GHSOM) was studied, the gray relational analysis was introduced into the GHSOM network, and the GRAGHSOM algorithm was proposed. The experimental results show that the GRAGHSOM algorithm reflects the importance of each component of the sample vector in the model in the process of high-dimensional data clustering and can perform clustering more accurately [2–5].

Because the fitting of a single neural network to a nonlinear dynamic system is generally computationally intensive, slow in convergence, time-consuming, and poor in generalization ability, a modular neural network chaotic parallel self-organizing network prediction method is proposed. That is, the phase space after the reconstruction of the chaotic parallel self-organizing network is divided into multiple subspaces, and an unbalanced subnet strategy is adopted. A neural network with a different structure is used in each subspace to calculate and track the local attractor trajectory, and the prediction result is finally passed into integrated module output [6]. This method not only makes use of the characteristics of self-organization and self-learning of neural networks but also greatly improves its computational performance compared with a single neural network. It can track the attractor trajectory more accurately in the subspace, thereby improving the overall prediction. Clustering refers to the grouping of objects in the database into meaningful subsets, so that the members in a class are as similar as possible, and the differences in members among different classes are as large as possible. Clustering has unsupervised learning capabilities and is widely used in many fields [7–10].

This article first reviews the commonly used BP neural network and self-organizing neural network in neural network models. Then referring to the article, a new self-organizing difference theoretical model was established on the basis of self-organizing, and a better neural network structure was found through comparison. A better model was found by comparing the number of different classes and the prediction error of the hidden layers. The reference article combines the self-organizing neural network with the autoregressive model, improves the weight initialization and learning rate of the neural network, and finds a model with less error by comparing the number of different categories. In the article, the BP neural network is selected as the comparison model to evaluate the effect of the new model. The single-even network and multi-even network forecast models of the forecast system are studied. Matlab 6.5 programming is used to train the breakout forecast system based on the nonlinear neural network, using the simple, practical, and easy-to-program Visual C++ software for interface design, and a visual forecasting system based on nonlinear neural networks was developed. Experiments have proved that the use of a breakout prediction system based on a nonlinear neural network for breakout prediction can improve the prediction accuracy.

## 2. Related Work

Therefore, in recent years, the prediction method of chaotic parallel self-organizing network based on a neural network has obtained preliminary theoretical and application research results. However, on the one hand, most of the current researches are focused on the optimization of the network structure itself and the improvement of the two independent processes of phase-space reconstruction parameter selection, without considering the two as a whole. The reconstructed phase umbrella is divided into subspaces in order to track the attractor's trajectory with higher precision locally.

Torrents et al. [11] believe that the phase space of the system can be reconstructed from the parallel self-organizing network of a variable, and all the information of the entire dynamic system is contained in the chaotic parallel self-organizing network itself. The embedding theorem of Grossberg [12] further proves the idea and provides a theoretical basis for predicting the chaotic time series. However, how to construct the prediction model according to the phase reconstruction theory, select the reconstruction phase-space parameters, and predict model parameters is a key problem in the prediction of chaotic parallel self-organizing networks. For this reason, many nonlinear prediction methods for predicting chaotic parallel self-organizing networks have been proposed. Among them, the neural network-based prediction method has distributed processing, self-organization, and self-adaptation. The excellent characteristics of self-learning columns and fault tolerance can better deal with uncertain and nonlinear prediction problems, so more research and applications have been obtained in the prediction of chaotic parallel self-organizing networks. Mohammadi et al. [13] used the neural network global method to predict the chaotic parallel self-organizing network. The results proved that when the MLP neural network is used as a global prediction method to predict the chaotic parallel self-organizing network, the effect is better than most local prediction methods. RBF networks have a strong biological background and are often used in the prediction of chaotic parallel self-organizing networks. Researchers are committed to improving the hidden layer node function and number selection of classic RBF networks.

The initial global prediction methods for chaotic parallel self-organizing networks mostly used feed-forward neural networks. There are many types of feed-forward networks, mainly including multilayer perceptrons (MLP) neural networks and radial basis functions neural network, nonlinear neural network (WNN), and fuzzy neural network (FNN). A representative MLP network is Park [14], which uses a multilayer feedforward network to simulate Lorenz chaotic sequences, where *m* represents phase-space reconstruction. The embedding dimension and its conclusion illustrate the important role of neural network in the prediction of chaotic time series. Yuan and Li [15] studied the gradient changes of the parallel self-organizing network. The experimental results show that the prediction performance of the classic RBF network has been improved [16]. The researchers proposed a cross-validation subspace method to select the optimal number of hidden layer nodes. This method is used in the prediction of noise-containing chaotic parallel self-organizing networks. The simulation object is the chaotic sequence of radar sea echoes. Nonlinear neural network is a new type of feedforward neural network based on nonlinear theory. Nonlinear neural network is compatible with the superiority of nonlinear and neural networks [17–20].

## 3. Construction of Sports Event Score Prediction Model Based on a Parallel Self-Organizing Nonlinear Neural Network

### 3.1. Parallel Self-Organizing Architecture Nesting

**Figure 1:**
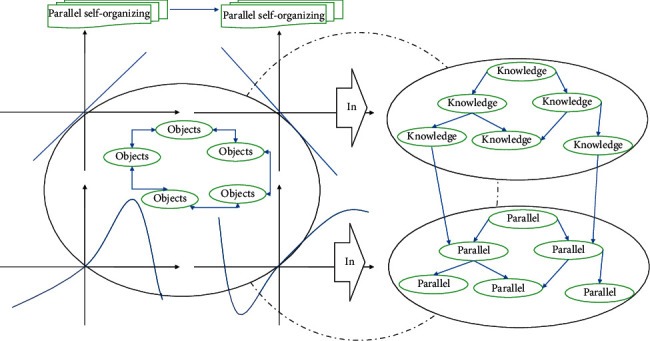
Parallel self-organizing architecture nesting.

According to the different objects to be studied, the behaviors of parallel self-organizing networks are very different. Even for the same object, there will be different performances at different times. Therefore, in order to treat different time series in different ways, parallel self-organizing networks can be classified according to the way of data selection and the form of data changes over time.  Figure 1 shows the nested parallel self-organizing architecture.

When using the combination of difference weighted average to predict the parallel self-organizing network, the self-organizing neural network to the historical data is first used and the input data are divided into several categories. The target value is calculated according to the formula to calculate the weighted average of the difference. When predicting the behavior of new data, first classify it according to the trained self-organizing neural network and then add the weighted average corresponding to the class of the data to the last value of the new data, which is the prediction corresponding to the new data.

(1) $$\begin{array}{c} R-\int_{0}^{\infty }\left(R_{FR}+R_{F1}-\frac{R_{FR}+R_{F1}}{R_{FR}R_{F1}}\right)\text{d}t=0, \end{array}$$

(2) $$\begin{array}{c} \exp\left[i\;df\left(w_{i}\right)\right]-\exp\frac{\left|D\right|}{\left|\left\{D_{i}:D_{j}\in w_{j}\right\}-1\right|}=0. \end{array}$$

First, we must establish a self-organizing neural network to classify the known data. The known data exist in the form of vectors. The elements of each vector are the parallel self-organizing network values selected from the parallel self-organizing network at a certain time interval. And all vector elements are selected in the same way. Let the dimension of the vector be *l*. The network weights of the initial self-organizing neural network are randomly selected. The risk value of the network is obtained by adding the risk value of the host

(3) $$\begin{array}{c} u\left(k,t,x\left(k,t\right)\right)=\left\{\begin{array}{cc} \varepsilon \left(i,n\right)\times w\left(k,t\right), & 0<t<k,\\ \delta \left(i,n\right)\times w\left(k,t\right), & t>k,\\ y\left(n\right)\times w\left(k,t\right), & t<0, \end{array}\right) \end{array}$$

(4) $$\begin{array}{c} \sum \nabla^{2}\frac{\alpha -1}{a^{2}}*\frac{\partial^{2}\alpha }{\partial t^{2}}+\int \frac{\rho }{\varepsilon }\text{d}x\text{d}y=0. \end{array}$$

When all the known data are input to the self-organizing neural network, the optimal neuron is obtained, and the weight vector of the optimal neuron and its surrounding neurons are adjusted, and the self-organizing neural network completes one step of training. Then, the data are re-input into the self-organizing neural network for the next step of training, until the optimal neuron corresponding to each input vector in the two trainings before and after does not change, and the self-organizing neural network completes the training. Taking the weight vector of the neurons in the competitive layer after training as the clustering center, the data can be classified finally.

(5) $$\begin{array}{c} f\left(x_{i},x_{2},x_{3}\right)=G\left(F\left(x_{1},W\left(x\right)\right)\right)G\left(F\left(x_{2},W\left(x\right)\right)\right)G\left(F\left(x_{3},W\left(x\right)\right)\right). \end{array}$$

The neurons receive the data passed by the neurons in the previous layer and adjust the data according to their own transformation functions. We perform the transformation and then pass the transformed data to the next layer of neurons. The last part receives the data of the last layer of hidden layer neurons and calculates the final result and error of this input.

### 3.2. Nonlinear Neural Network Node Output

**Figure 2:**
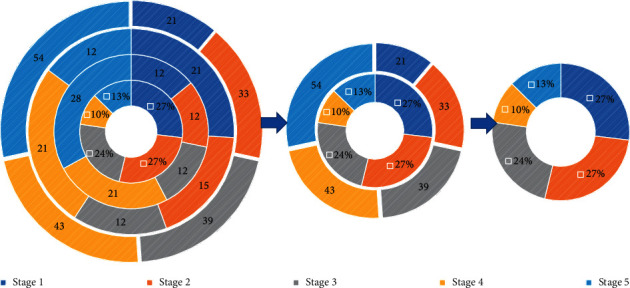
Node distribution of the nonlinear neural network.

Structurally, the characteristics of nonlinear neural networks are as follows: (1) distributed storage: that is, all neurons of the neural network jointly complete the information processing function, and each neuron has an independent function to process data; (2) parallelism: that is, the ability of all neurons of the neural network to complete information processing concurrently; and (3) fault tolerance: a single neuron's error in information processing will not affect the entire system. In terms of ability, the neural network can spontaneously react to external input and output data and makes appropriate adjustments to its own network parameters according to the input and output samples to adapt to the new input samples. Each neuron in the input layer stores an element of the input vector, and all neurons assign data values to the discriminant function of the neurons in the competition layer through the connection with the data according to the discriminant function, select the best matching neuron by comparing the value of the discriminant function of each neuron, and modify the network connection weight parameter according to the input data. The connections represent a certain distance between neurons in the competition layer. The distribution of this weight in one or two dimensions is fixed and will not change during training.  Figure 2 shows the distribution of nodes in a nonlinear neural network.

In a forward network without feedback, once a signal passes through a neuron, the neuron's processing process ends. In an interconnected network, signals have to be repeatedly transmitted between neurons, and the network is in a dynamic state of constant change. Starting from a certain initial state, after several changes, it will reach a certain equilibrium state. According to the structure of the network and the characteristics of neurons, the operation of the network may also enter periodic oscillations or other equilibrium states such as chaos. This parallel self-organizing network can also be called the aggregate index parallel self-organizing network, which is a statistical sequence arranged in time, and the data content is the statistical value of the same phenomenon or characteristic value. From the absolute parallel self-organizing network, the overall level of the phenomenon to be studied in a certain period can be observed, and it can also reflect the level of the phenomenon at a certain point in time. Therefore, the absolute parallel self-organizing network can be further divided into time series and time-point series according to the time selection method. The changes in the value of the curvilinear parallel self-organizing network are also inclined, but the tendency is not fixed, the magnitude and direction of the change may change, and the change of the value is not periodic. The linear parallel self-organizing network value changes have a certain tendency; that is, within a long time range, the value of the parallel self-organizing network gradually rises or decreases, and an upward or downward trend can be observed.

### 3.3. Quantification of Score Prediction in Sports Events

**Figure 3:**
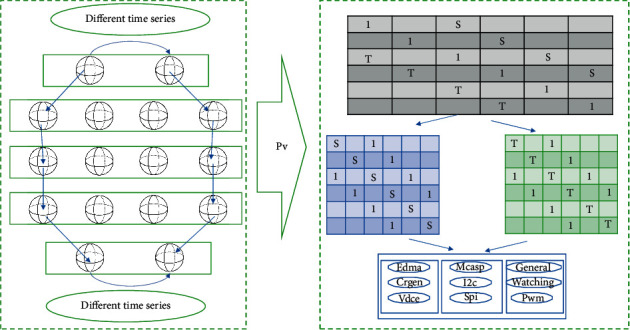
Quantitative processing of score prediction for sports events.

The normalization of artificial neural network sports score prediction requires proper processing of data to meet the special requirements of artificial neural network for data. According to different neuron transfer functions, many artificial neural network models only accept numerical data in the range of [0, 1] and [−1, 1]. Therefore, the data must be scaled down to this interval. Scalar numerical data are generally evenly distributed in a certain range and can be directly mapped to the interval [0, 1]. If the distribution of numerical data is not uniform, you can use piecewise linear equation or logarithmic equation to transform the data, and then scale down *d*, *N* to specify the interval. Discrete data are represented by encoding them with 0 and 1, or assigning a value to them in a specified continuous interval. Numerical data represented by vectors or arrays can sometimes be processed in groups; that is, the vector as a whole is subjected to regularization processing. There are several regularization methods, the most commonly used is to calculate the square root of the sum of the squares of the elements, and then it is used to remove each element. The second method is to first find the sum of all the elements, and then it is used to divide each number. In this case, the sum of the elements after regularization is 1.0, and the value of each element represents their contribution to the group. The third method is to remove each element with the maximum value in the vector.  Figure 3 shows the quantitative processing of score prediction for sports events.

Therefore, all of the above factors can be converted into time factors, and the parallel self-organizing network analysis method can be used to mathematically model the time factor, that is, the value of the score of a sports event. Through the historical value of the score of a sports event, it is possible to discover the change of the score of a sports event over time and predict the future development of sports event scores. On the other hand, the score of sports events is a relatively open data, which is relatively easy to obtain, which also provides convenience for research. The parallel self-organizing network of sports scores is a parallel self-organizing network with the value of sports scores as data elements. The scores of sports events can be taken every hour, every day, or other time as the parallel self-organizing network of sports scores. In competition, a vector is used as the input of the neural network. Each unit of the competition layer will form a discriminant function according to the weight of its own network and calculates the discriminant function with the input vector as the value of the independent variable, and the function value is the best. The neuron becomes the best matching neuron in this input. Other neurons surrounding the best matching neuron within a certain range will participate in the cooperation and become the neuron to be adjusted in the process of self-adaptation. Adaptive neurons within the topological neighborhood of the optimal neuron must modify their own network connection weights according to the value of the input vector to achieve the discriminative mode of these neurons.

### 3.4. Recursive Prediction Model Weight Factor

Data preprocessing is an important step in the data prediction process. The quality of the data directly affects the quality of the mining effect. Data preprocessing includes data cleaning, data integration, data conversion, and data reduction. The task of data cleaning is to eliminate noise or inconsistent data. The inconsistency of the data will reduce the credibility of the data prediction results.

**Figure 4:**
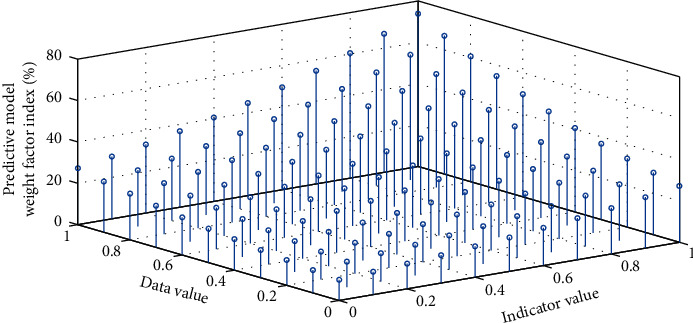
Weight factor clustering of the forecasting model.

The risk value of the host is obtained by adding the risk value caused by the vulnerabilities of the related state nodes. The database construction module is responsible for processing the system configuration information and vulnerability information obtained by the scanning module, extracting their nonquantitative attributes and quantifiable attributes, forming an information database and a vulnerability database; it is also responsible for analyzing the relationship among vulnerabilities. Mining was performed to form a vulnerability database. The data used for data prediction may come from multiple actual systems, so there is a problem of heterogeneous data conversion. Because there may be many inconsistencies between the data of multiple data sources, data integration is not a simple copying process. Data transformation is the transformation or unification of data into a form suitable for mining, including data generalization, normalization, attribute construction, and smoothing. Data reduction includes operations such as data aggregation, attribute selection, and data compression.  Figure 4 shows the clustering of the weight factors of the prediction model.

Multilayer perceptron is a popularization form of the single-layer perceptron. The information of the multilayer perceptron network is forwarded layer by layer, and each unit of the lower layer is connected to each unit of the upper layer. The input unit operates layer by layer according to the input/output relationship, and the connection weight between each layer can be adjusted by learning rules. It can be seen that the multilayer perceptron is actually a design of multiple single-layer perceptrons through appropriate combination, which can realize any shape division. In this method, each discrete class is assigned a value from 1 to *N* represented by a binary number. For example, if a variable has 32 possible values, it can be represented by a binary vector of length 5. As long as the discrete value is arbitrary and does not have any order, binary code is a better representation. However, when the discrete value is converted to a binary code, the bit value varies greatly. For example, the binary code of the seventh element in a discrete class is 000111, and the binary code of the eighth element is 001000. Hamming distance is a measure of the similarity between two binary numbers, expressed by the number of bits of different symbols in two codes. In this case, the Hamming distance between 7 and 8 is 4. If you want the neural network to think that the two input modes of 7 and 8 are similar, you must choose the thermometer code.

## 4. Application and Analysis of Sports Event Score Prediction Model Based on Parallel Self-Organizing Nonlinear Neural Network

### 4.1. Parallel Self-Organizing Nonlinear Neural Network Data Preprocessing

**Figure 5:**
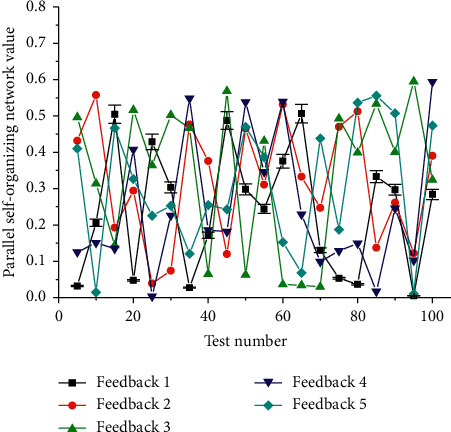
Distribution of connection points of parallel self-organizing nonlinear neural network.

The nonlinear neural network selected in this paper has a hidden layer, and every 10 pieces of data are used as a set of inputs. In the training phase, a matrix composed of 500 groups of 10-dimensional input vectors is selected as the input matrix, and the matrix composed of the target value corresponding to each group of input vectors is used as the output matrix. The parameters are adjusted until the error reaches the target value of training times, stop training. In the prediction stage, the new data are used as the input vector, the corresponding predicted target value is calculated according to the adjusted BP neural network, and the actual target is compared to evaluate the performance in Figure 5.

For the measured parallel network, we calculate the ratio of pseudo-neighbors from the minimum embedding dimension. When the ratio of embedding dimension m to pseudo-neighbors is less than 5%, pseudo-neighbors no longer increase with embedding dimension. When it decreases, it can be considered that the singular attractor is fully expanded, and *m* at this time is the minimum embedding dimension. Generally speaking, when the distribution of phase-space points is relatively sparse, it is better to use the pseudo-neighbor point method, because this method can automatically adjust the distance scale with the sparse changes of the phase-space points so that the statistical results are more reliable. When the phase-space points are densely distributed, it is more reasonable to use the saturated correlation dimension method.

To quantify the risk value of the state node, it is necessary to calculate the probability that the attacker can successfully attack to reach each state node. It can be seen that the average relative error of the PCA-GABP prediction model is both the smallest. The number less than 3% is 39, which is 2 more than the number where the absolute value of the relative error of the GABP model is less than 3%. After comparing and analyzing the data in the article, it can be concluded that the model whose input data have been processed by PCA has a higher prediction accuracy than the unprocessed model; at the same time, regardless of whether the input data has been processed by PCA, the BP neural network improved by GA prediction accuracy is significantly higher.

### 4.2. Realization of the Simulation of the Score Prediction Model for Sports Events

Also taking the sports event factor as an example, the GA nonlinear neural network has 48 inputs; that is, there are 48 neurons in the input layer of the network, and the output is also 48; that is, there are 48 neurons in the output layer of the network. Using the floating-point coding scheme, each connection weight or threshold is directly represented by a real number, and each individual contains a network ownership value and threshold. The weight of a network is composed of two parts.

**Figure 6:**
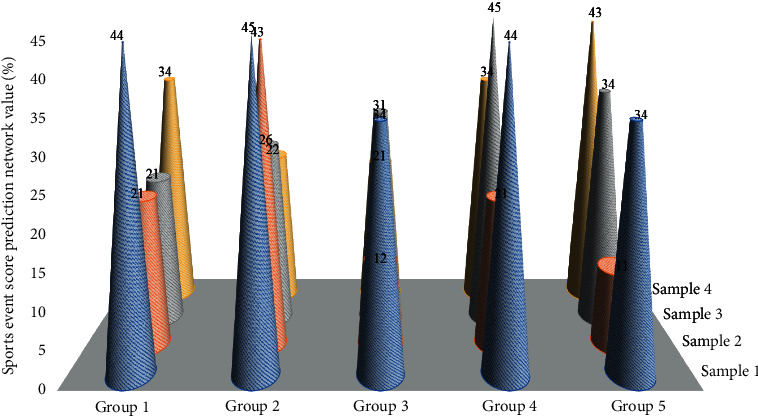
Score prediction and training situation of sports events.

Finally, the asset damage value caused by the related vulnerabilities is multiplied by the asset value of the host node to quantify the risk. This article has 48 input neurons and 48 output neurons. Assuming there are 36 hidden layers, plus the thresholds of the hidden layer and the output layer, the code length of each individual is 48×36 + 36 × 48 + 36 + 48 = 3540. We input the training samples in the article into the prediction model, and after many debugging, it is finally determined that there are 36 nodes in the hidden layer. The simulation results of the GABP neural network model are as described in the text.  Figure 6 shows the score forecasting training situation of sports events.

If the total input of the neuron is far away from the threshold, the actual output of the neuron is either the maximum value or a small value due to the saturated nonlinear characteristics of the neuron. When the total input of a neuron is too far above the threshold, it is said to enter the saturation zone. When the total input falls into the saturation zone and the actual output contradicts the target output, it is incorrect saturation, that is, false saturation. At this time, there needs to be a large amount of modification to the weight, but in fact, because the derivative value is close to zero at this time. The amount of modification of the derivative weight is very small. The same is true for hidden layer neurons, but the error is calculated by error back propagation. Once learning enters the false saturation state, it will take a long time to get rid of this state or even unable to find the global optimum.  Table 1 is the description of hidden layer neuron training.

Calculated by the pseudo-neighbor method, the minimum embedding dimension *m* is 9, the DRNN model is constructed, the recursive delay is 1, and the same 9 × 21 × 1 nonlinear neural network is constructed. The first 2000 points among the 3000 points are used as training data, and the last 1000 points are used as prediction data. Since the predicted curve is relatively close to the actual curve, it is difficult to distinguish it from the graph, so only the absolute error *P*(1) of the DRNN model and the nonlinear neural network prediction are given.

When network administrators find unknown vulnerabilities, they need to integrate the vulnerability information and submit it to the national information security vulnerabilities by e-mail. In the training phase, the network is used to layer the input data into several categories and calculate the weighted average difference corresponding to each category of data. In the prediction stage, the trained neural network is used to divide each group of input data into a certain classification area, and then, the prediction is made based on the calculated weighted average difference. The article lists the results of the self-organizing difference weighted average method to predict the results of the parallel self-organizing network of the sports event score, that is, when the number of historical data classifications is different.

### 4.3. Example Application and Analysis

In the experiment, we set the output as *y*(*t* + 1) and then input as *x*(*t* − 4). The network has 5 nodes, and the time interval between each neuron is 1. In the experiment, 2500 data are generated on each chaotic parallel self-organizing network of the benchmark, and the whole of them is divided into three parts: training data, verification data, and test data. The ratio of the amount of data of the three parts is 14 : 6 : 5, that is, 1,400 training data, 600 verification data, and 500 prediction data. It can be seen that the hidden layer nodes should be twice the input layer, that is, 10 nodes.

**Figure 7:**
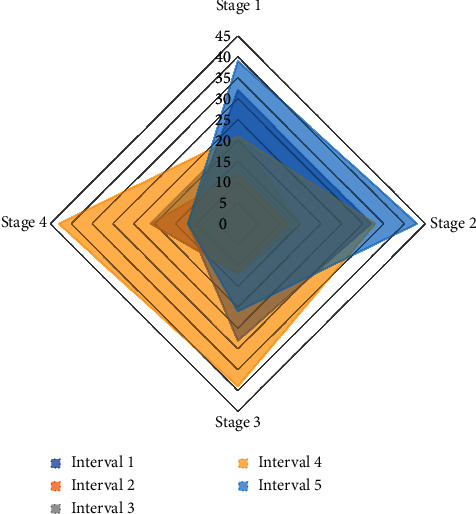
The output effect of the sports event score prediction network.

Then, the staff will analyze the vulnerabilities, and only after several working days can the information such as the category and hazard value of the vulnerabilities, be published on the official vulnerability platform. The output layer has only one neuron, that is, the predicted value of the stock closing index on the next day. The structure of the recurrent neural network is 51. The DRNN model in this paper is greatly improved relative to the nonlinear neural network model, and it can be seen that the fitting degree of the true exponential curve is also better than the fitting degree of the nonlinear neural network model. It can be seen that the error of the recursive model in this article is concentrated between (−20, 20), the error value is small, the error of the nonlinear neural network model is between (−80, 40), the error value is large, and some points deviate greatly from the true value, which is caused by the high degree of the feedforward network.  Figure 7 shows the output effect of the sports event score prediction network.

**Figure 8:**
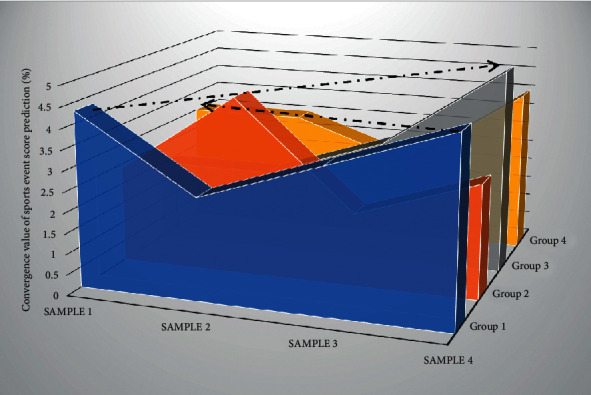
Convergence of the sports event score prediction network.

This will not only cause a huge workload for the vulnerability management staff, but, more importantly, the network administrators will not be able to obtain information about unknown vulnerabilities in a timely manner. Because these feature variables may have high correlations, these features are redundant features. The results show that although the overall detection rate is very high, the U2R class is almost undetectable, and the R2L detection rate is relatively low. There is no doubt that this is because the number of samples is too small, which makes it difficult to build a classifier for these attack classes. The error detection rate is high, and the two types are mostly detected as normal types, which are absolutely not allowed in IDS. To avoid this, deduplication is performed before training. In order to improve the performance of the classifier without greatly changing the number of samples in the data set, this article increases the number of U2R samples in the data set to ensure the basic classification performance. The system structure adopts the client/server structure. The server is responsible for scanning the hosts and devices in the network to obtain the configuration information and vulnerability information of all the hosts in the network; the client is responsible for collating the scanning results to form various databases. On this basis, certain mathematical methods are used to measure the overall risk assessment value of the network and determine the risk level. Therefore, in addition to filtering irrelevant features, the optimal feature subset must also filter redundant features.  Figure 8 shows the convergence of the sports event score prediction network.

There are *N* individual individuals to form the initial population. The value of the population number *N* will affect the efficiency of the entire algorithm. If the value of *N* is too large, the calculation amount will increase, and the running speed will be too slow. If the value of *N* is too small, although the operating speed can be increased, the diversity of the population is reduced to a certain extent, which may cause premature convergence, that is, premature maturity. According to experience, the value of *N* is generally between 10 and 200.

Feature selection methods can affect machine learning performance, model complexity, and model running speed. One of the more popular feature selection methods is to select *n* variables that are highly correlated with categorical variables. Therefore, only the first six principal components need to be extracted under the condition that the loss of the original data is not too much information, so as to achieve the purpose of principal component extraction. It can be seen that the prediction points with larger relative errors are significantly reduced, which is 0.5% less than the maximum relative error of the nonlinear neural network, After taking the absolute value of 48 relative errors, 4 points have an error of more than 5%, 4 points are between 4% and 5%, 3 points are between 3% and 4%, and 8 points are between 2% and 2%, and the improved BP neural network has achieved better prediction results.

## 5. Conclusion

By reviewing relevant materials and literature, we outline data prediction and neural network related research. The artificial neural network data prediction, data transformation, data representation, and data preprocessing are studied. We made a more in-depth study on the use of artificial neural network methods for clustering, based on the in-depth study of the SOM clustering algorithm, optimized the SOM learning algorithm, and then studied the layer expansion self-organizing mapping network (GHSOM), and put forward the GRAGHSOM algorithm that introduces gray relational analysis. Regional competitiveness is a typical nonlinear complex giant system, and it is difficult for traditional linear methods to obtain satisfactory prediction results. At the same time, the experiment proves that this model has great advantages in pattern recognition. The hidden layer quantum neuron of the nonlinear model uses the linear superposition of the nonlinear basis function as the activation function. At the same time, the thesis gives the learning algorithm of a nonlinear neural network. However, in feature selection, combining variables that are highly correlated with categorical variables may not necessarily improve the performance of the classifier. The neural network method can handle systems that are difficult to describe with mathematical models. It has strong parallel processing, self-adaptation, self-organization, and arbitrary approximation to nonlinearity. The experimental results show that the GRAGHSOM algorithm reflects the importance of each component of the sample vector in the model in the process of high-dimensional data clustering and can perform clustering more accurately; for the application of local sports score prediction (LWF) in training, we studied the perceptron neural network and successfully applied the perceptron neural network to the local sports event score prediction system.

## Figures and Tables

**Table 1:** Description of hidden layer neuron training.

Index number	First level	Second level	Third level
1	DRNN layer capability	0.31	0.64
		0.22	0.21
		0.54	0.08
2	DRNN layer ability	0.24	0.13
		0.46	0.56
		0.11	0.34
3	Hidden layer neuron training	0.09	0.24
		0.27	0.32

## Data Availability

The data used to support the findings of this study are available from the corresponding author upon request.

## References

[1] Zhang W., Zhang R., Shang R. (2019). Application of natural computation inspired method in community detection. *Physica A: Statistical Mechanics and Its Applications*.

[2] Ruan L., Li C., Zhang Y., Wang H. (2019). Soft computing model based financial aware spatiotemporal social network analysis and visualization for smart cities. *Computers, Environment and Urban Systems*.

[3] Ramos-Villagrasa P. J., Marques-Quinteiro P., Navarro J., Rico R. (2018). Teams as complex adaptive systems: reviewing 17 years of research. *Small Group Research*.

[4] Hao Y., Miao Y., Chen M., Gharavi H., Leung V. C. M. (2021). 6G cognitive information theory: a mailbox perspective. *Big Data and Cognitive Computing*.

[5] Metcalf L., Askay D. A., Rosenberg L. B. (2019). Keeping humans in the loop: pooling knowledge through artificial swarm intelligence to improve business decision making. *California Management Review*.

[6] Hristovski R., Balagué N. (2020). Theory of cooperative-competitive intelligence: principles, research directions, and applications. *Frontiers in Psychology*.

[7] Kong F., Li J., Wang Y. (2019). Human-computer interactive teaching model based on fuzzy set and BP neural network. *Journal of Intelligent and Fuzzy Systems*.

[8] Xu X., Ye Z., Li J., Xu M. (2018). Understanding the usage patterns of bicycle-sharing systems to predict users’ demand: a case study in Wenzhou, China. *Computational Intelligence and Neuroscience*.

[9] Schmit C., Brisswalter J. (2020). Executive functioning during prolonged exercise: a fatigue-based neurocognitive perspective. *International Review of Sport and Exercise Psychology*.

[10] Maksakova O., Zhavoronkova L. (2020). Transdisciplinary team in rehabilitation of unconscious brain-damaged persons: grounds and practice. *Journal of Behavioral and Brain Science*.

[11] Torrents C., Balagué N., Ric Á, Hristovski R. (2020). The motor creativity paradox: constraining to release degrees of freedom. *Psychology of Aesthetics, Creativity, and the Arts*.

[12] Grossberg S. (2021). Attention: multiple types, brain resonances, psychological functions, and conscious states. *Journal of Integrative Neuroscience*.

[13] Mohammadi M., Al-Fuqaha A., Sorour S., Guizani M. (2018). Deep learning for IoT big data and streaming analytics: a survey. *IEEE Communications Surveys & Tutorials*.

[14] Park C. (2021). Dynamic coordination phase and joint profiles: cluster and fixed-point shift techniques. *International Journal of Applied Sports Sciences*.

[15] Yuan H., Li G. (2021). A survey of traffic prediction: from spatio-temporal data to intelligent transportation. *Data Science and Engineering*.

[16] Gupta S., Kaur M., Lakra S., Dixit Y. (2020). A comparative theoretical and empirical analysis of machine learning algorithms. *Webology*.

[17] Yang R., Wang D. (2020). Hierarchical aggregation for reputation feedback of services networks. *Mathematical Problems in Engineering*.

[18] Calvo Tapia C., Villacorta-Atienza J. A., Díez-Hermano S. (2020). Semantic knowledge representation for strategic interactions in dynamic situations. *Frontiers in Neurorobotics*.

[19] Buszard T., Garofolini A., Reid M., Farrow D., Oppici L., Whiteside D. (2020). Scaling sports equipment for children promotes functional movement variability. *Scientific reports*.

[20] Bolfíková E., Pirohová I., Lenhardtová M. (2019). Bureaucracy and creative complexity–empirical analysis of problem solving effectiveness within organizations. *Sociologija i Prostor: Časopis Za Istraživanje Prostornoga I Sociokulturnog Razvoja*.

